# Electrodeposited Ionomer Protection Layer for Negative Electrodes in Zinc–Air Batteries

**DOI:** 10.3390/membranes13070680

**Published:** 2023-07-20

**Authors:** Papa K. Kwarteng, Suanto Syahputra, Luca Pasquini, Florence Vacandio, Maria Luisa Di Vona, Philippe Knauth

**Affiliations:** 1 Aix Marseille Univ, CNRS, MADIREL (UMR 7246), Electrochemistry of Materials Group, Campus St Jérôme, 13013 Marseille, France; papa-kwakye.kwarteng@etu.univ-amu.fr (P.K.K.); suanto.syahputra@etu.univ-amu.fr (S.S.); luca.pasquini@univ-amu.fr (L.P.); florence.vacandio@univ-amu.fr (F.V.); 2 Tor Vergata University of Rome, Department Industrial Engineering, Via del Politecnico 1, 00173 Roma, Italy; divona@uniroma2.it

**Keywords:** metal–air batteries, oxygen reduction reaction, anion exchange membranes, electropolymerization

## Abstract

The protection of zinc anodes in zinc–air batteries (ZABs) is an efficient way to reduce corrosion and Zn dendrite formation and improve cyclability and battery efficiency. Anion-conducting poly(N-vinylbenzyl N,N,N-trimethylammonium)chloride (PVBTMA) thin films were electrodeposited directly on zinc metal using cyclic voltammetry. This deposition process presents a combination of advantages, including selective anion transport in PVBTMA reducing zinc crossover, high interface quality by electrodeposition improving the corrosion protection of zinc and high ionomer stiffness opposing zinc dendrite perforation. The PVBTMA layer was observed by optical and electron microscopy, and the wettability of the ionomer-coated surface was investigated by contact angle measurements. ZABs with PVBTMA-coated Zn showed an appreciable and stable open-circuit voltage both in alkaline electrolyte (1.55 V with a Pt cathode) and in miniaturized batteries (1.31 V with a carbon paper cathode). Cycling tests at 0.5 mA/cm^2^ within voltage limits of 2.1 and 0.8 V gave a stable discharge capacity for nearly 100 cycles with a liquid electrolyte and more than 20 cycles in miniaturized batteries. The faster degradation of the latter ZAB was attributed to the clogging of the carbon air cathode and drying or carbonation of the electrolyte sorbed in a Whatman paper.

## 1. Introduction

Metal–air batteries are assembled from a metal anode and a porous cathode in a suitable electrolyte [1,2,3]. They combine the design of fuel cells and conventional batteries and have been demonstrated to have large theoretical energy densities: zinc–air batteries (ZABs) have a theoretical energy density of about 1350 Wh/kg [4,5] which is higher compared to devices based on Li-ion chemistry, due to the ability to use air at the cathode and to exchange two electrons per zinc atom. ZABs represent a safe, environmentally benign, affordable and simple way to store and deliver electrical energy for a wide variety of devices [6]. However, research efforts are necessary to increase the durability of rechargeable ZABs and mitigate obnoxious occurrences that decrease the number of charge–discharge cycles such as zinc corrosion and dendrite formation, cathode degradation and alkaline electrolyte carbonation [7,8,9].

During the discharge of the battery, the chemical reaction scheme below captures the main processes involved in concentrated KOH [10].

Anode:

(1) $$Zn+{4OH}^{-}⇆{Zn\left(OH\right)}_{4}^{2-}+2e^{-}\;\;\;\;E{}^{\circ}=-1.26\;V\;vs.\;SHE$$

Cathode:

(2) $$\frac{1}{2}O_{2}+2e^{-}+H_{2}O⇆2OH^{-}\;\;\;\;E{}^{\circ}=0.40\;V\;vs.\;SHE$$

Concerning the oxygen reduction and evolution reactions at the positive electrode, a highly efficient catalyst layer with high catalytic activity, high stability and optimal mass transport properties greatly increases the overall efficiency of a ZAB [3,11,12,13,14]. Although some innovative metallic catalysts have been proposed recently [15,16,17], the goal is to propose more environmentally friendly metal-free electrode materials with appreciable catalytic activity for oxygen reduction and evolution. At the current state of the art, transition metal oxides are developed as the best-performing catalysts; however, inexpensive carbon material support improves the catalyst dispersion and active area and decreases the cost [18]. Doped carbon materials can also be potent electrocatalysts, especially for the oxygen reduction reaction in alkaline conditions [8,12,19,20,21,22,23]. Problems arise also concerning the shape and structural changes of the zinc electrode during repeated charge/discharge cycles [3]. A major challenge is the spontaneous reaction between Zn and the alkaline electrolyte which can generate hydrogen. Active material is lost in such instances, which affects the overall efficiency; the zinc electrode corrosion leads finally to cell failure. Furthermore, the existence of nonuniform current distribution and concentration gradients in the electrolyte causes uneven zinc dissolution and re-deposition, which leads to dendrite formation and short-circuiting [6,9,24]. To counter the problem of zinc dendrite formation, current research is focused on the thin-film surface coating of the zinc electrode that reduces corrosion, keeps Zn^2+^ discharge products near the surface and protects them from being swept away into the electrolyte. Many coatings have been reported over the last years [25,26,27]. Furthermore, innovative electrolyte compositions, including, for example, chitosan [28] or nanocellulose [29], can significantly improve the cyclability of ZABs.

**Scheme 1:**
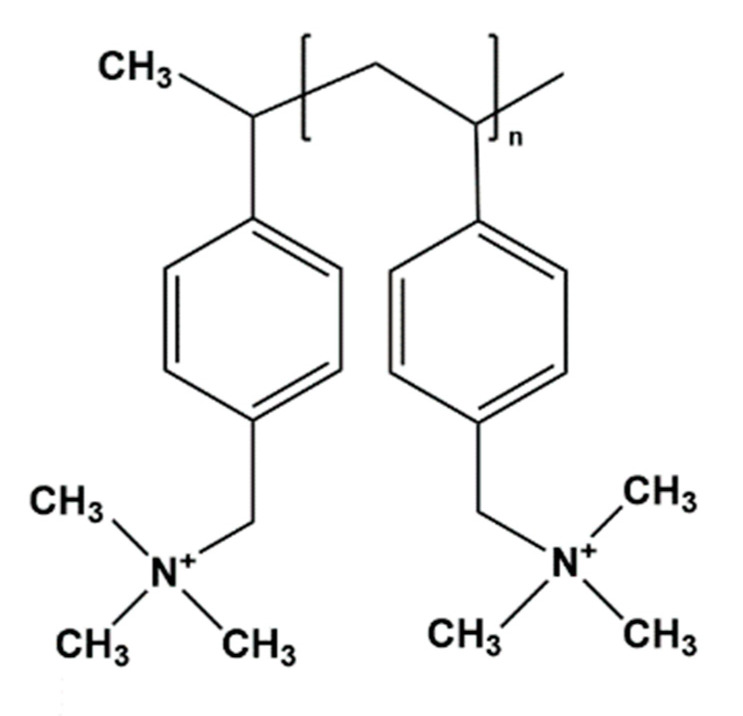
Chemical formula of PVBTMA.

In this work, we explore an advanced coating of the zinc anode by anion-conducting poly(N-vinylbenzyl N,N,N-trimethylammonium) chloride (PVBTMA, Scheme 1) that is electropolymerized [30,31] directly on the zinc metal. The major advances foreseen by this approach are the excellent quality of the metal/ionomer interface, due to the in situ electropolymerization process, which should improve the corrosion protection and the selective anion permeability of the coating, allowing the transport of small hydroxide ions but restricting the diffusion of larger zincate ions. In fact, Zn deposition in the presence of quaternary ammonium salts and polyelectrolytes was recently studied to improve cyclability by suppressing dendritic growth [32]. Furthermore, PVBTMA presents high stiffness and strength and should be capable of resisting the perforation by zinc dendrites, due to the polystyrene backbone. Finally, the polyelectrolyte separator facilitates the device miniaturization [33,34]. The resulting ZAB batteries with PVBTMA-coated Zn anodes are analyzed in terms of cyclability in an alkaline electrolyte and in solid-state configuration.

## 2. Materials and Methods

### 2.1. Electrodeposition of PVBTMA on Zinc Metal

Zinc pellets with a diameter of 15 mm (Neyco, 99.99%, Vanves, France) were polished using P800 polishing paper and water with ESC 200 GTL polisher (Escil, Chassieu, France) until the surface of the pellet was uniformly clean and smooth. The monomer solution for the electropolymerization of N-vinylbenzyl-N,N,N-trimethylammonium chloride (VBTMA, 99%, Sigma-Aldrich, Milano, Italy) on zinc pellets was prepared by dissolving 0.145 g of VBTMA (0.01) and 1.88 g of LiClO_4_ (0.25 M, 99.99%, Sigma-Aldrich, St Quentin Fallavier, France) in 70 mL DMSO (99.9%, Sigma-Aldrich, St Quentin Fallavier, France). A three-electrode setup was used for the electropolymerization with a Ag/AgCl (KCl saturated) reference electrode (E = 0.197 V vs. SHE). The PVBTMA coatings were deposited by cyclic voltammetry (CV) performed in a potential range between −1.0 V and −2.1 V vs. Ag/AgCl with a scan rate of 20 mV/s during 100 cycles. The counter electrode was a 4 cm^2^ Pt foil. All electrochemical experiments were performed by using a potentiostat-galvanostat VSP-300 (BioLogic, Seyssinet-Pariset, France).

### 2.2. Characterization

The polished zinc pellets before and after PVBTMA coating were imaged using a Leitz Aristomet microscope (Paris, France), and images were captured with a Euromex CMEX-5 camera (Arnhem, The Netherlands). The PVBTMA film thickness was measured with a Mitutoyo 293–230 micrometer (Roissy, France).

^1^H NMR experiments were performed using a Bruker Avance III operated (Milan, Italy) at 700 MHz using deuterated solvent (DMSO-d_6_). Chemical shifts (ppm) were referenced to tetramethylsilane. FTIR spectra were recorded with a Perkin Elmer Spectrum Two (Villebon-sur-Yvette, France) equipped with an ATR crystal diamond module in a wavenumber range of 500–4000 cm^−1^ with a resolution of 0.5 cm^−1^.

Scanning electron microscopy (SEM) micrographs were made with a high-resolution Zeiss Gemini 500 apparatus (Paris, France) at 5 kV acceleration voltage.

The contact angle measurements were performed using a Biolin Scientific Attension Theta Flex (Västra Frölunda, Sweden) optical tensiometer. Sessile drop experiments with water as the heavy phase (3 μL drops at 0.1 μL/s rate) and air as the light phase were used for Young–Laplace analysis.

### 2.3. Electrochemical Tests of Zn–Air Batteries

Potentiostatic electrochemical impedance spectroscopy (PEIS) was run on all samples using a BioLogic VSP-300 apparatus (Grenoble, France). The AC signal with an amplitude of 20 mV was scanned from 1 Hz to 1 MHz with a record of 10 points per decade in logarithmic spacing. PEIS was recorded until a stable plot was obtained for all samples due to the swelling ability of electrodes containing an anion exchange ionomer (AEI). The impedance spectra were analyzed using the ZFit EC-lab software by defining an equivalent circuit containing resistance and constant-phase elements (CPEs). The Zn–air battery performances were investigated by cycling tests in 6 M KOH containing 0.2 M ZnO in two-electrode configuration with a platinum counter electrode; the alkaline solution was saturated with oxygen by O_2_ gas bubbling at 0.2 L/min. A galvanostatic method was applied with constant current of 0.5 mA for charge and discharge during a set time of 20 min for each cycle. Voltage limits of 2.1 V and 0.8 V were set for charging and discharging, respectively. All solid-state Zn–air batteries were constructed by modifying a basic fuel cell setup purchased from Fuel Cell Store (Bryan, TX, USA). The ZAB includes a PVBTMA-covered Zn pellet as negative electrode, a Whatman paper (Paris, France) soaked in 6 M KOH and 0.2 M ZnO solution as separator and a positive electrode using porous acid-treated carbon paper (AvCarb EP55, Bryan, TX, USA) [18] exposed to air.

## 3. Results

### 3.1. Electropolymerization of PVBTMA on Zinc

**Figure 1:**
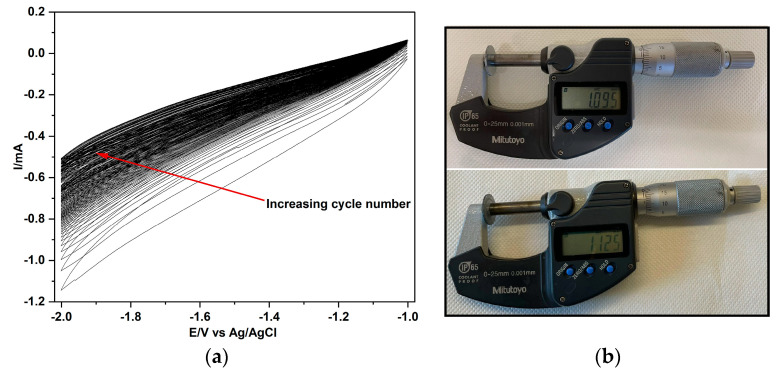
(**a**) Cyclovoltammogram of PVBTMA electrodeposition on Zn; (**b**) micrometric determination of PVBTMA coating thickness.

Figure 1 shows a typical cyclovoltammogram of PVBTMA deposition on a polished Zn disk. The decrease in the current with the cycle number can be attributed to the increase in the ionomer thickness and the related increase in sample and interfacial resistances (see the discussion of impedance spectra). The electropolymerization mechanism [30,31] involves a one-electron reduction with the cleavage of the vinylic double bond and formation of a radical anion, which forms a secondary radical by reaction with a proton (from residual water in hygroscopic DMSO). The secondary radical is the initiator of radical polymerization giving the polymer (Scheme 1).

**Figure 2:**
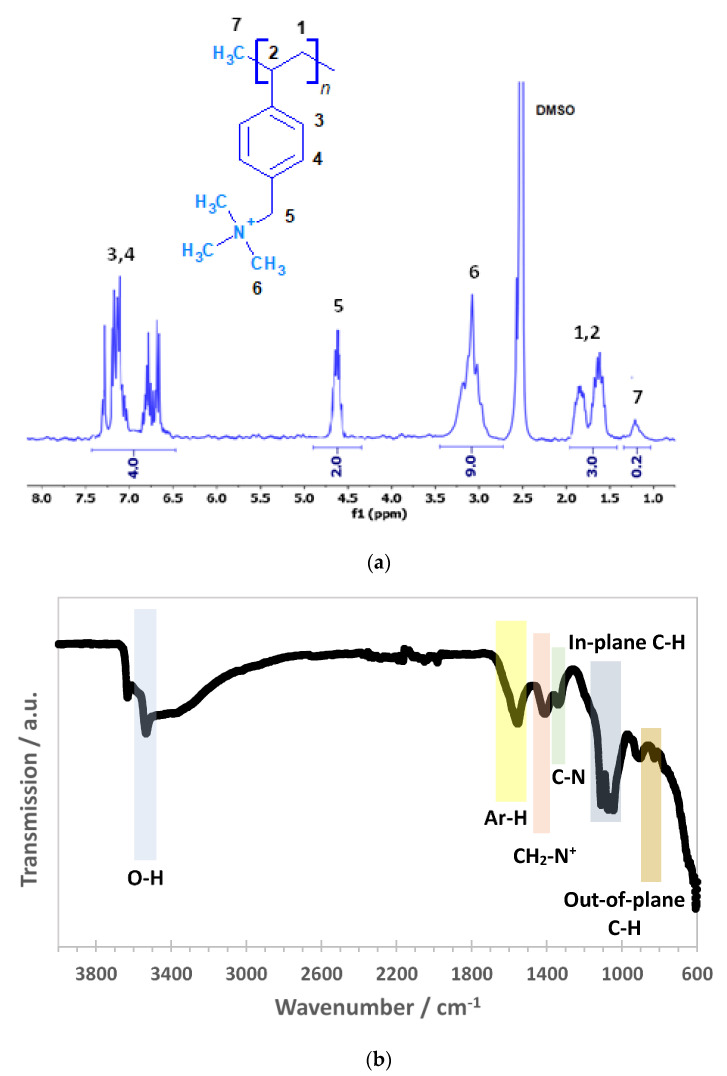
Spectroscopic characterization of PVBTMA. (**a**) ^1^H NMR spectrum; (**b**) FTIR spectrum.

The spectroscopic characterization of the ionomer layer is reported in Figure 2. The ^1^H NMR spectrum (Figure 2a) shows the peaks of the phenyl protons (3 and 4, 4H) in the region between 6.5 and 7.5 ppm. The signal centered at 4.6 ppm (5, 2H) is typical of benzylic protons near the ammonium moiety. The methyl group of trimethylammonium (6, 9H) around 3.0 ppm is clearly present in the polymer. The aliphatic signals of the polymeric backbone (3H) appear in the region between 1.1 and 1.9 ppm. The peak around 1.2 ppm (7) may be ascribed to the terminal methyl group of the polymer. From the ratio of peak 7 and peaks 3 and 4, taken as a reference (4H), the polymer has a length of about 15 monomer units.

The FTIR spectrum (Figure 2b) shows the relevant signals of PVBTMA, including a peak at 609 cm^−1^ ascribed to C-H out-of-plane bending. The peaks at 828 and 904 cm^−1^ can be attributed to the out-of-plane vibrations of aromatic C-H. Three peaks at 1046, 1073 and 1112 cm^−1^ correspond to the in-plane deformation of the aromatic ring. In addition, there are peaks at 1340 cm^−1^ ascribed to C-N stretching and 1410 cm^−1^ indicating the presence of CH_2_ of quaternary ammonium. A peak at 1555 cm^−1^ is ascribed to the vibration of aromatic moieties (in-ring). A small signal around 3000 cm^−1^ is characteristic of methyl C-H stretching. Two peaks around 3600 cm^−1^ correspond to OH free stretching, due to sorbed water by the hydrophilic ionomer. There are no double bonds observed around 1680–1640 cm^−1^ showing that the precursor was fully transformed.

The thickness *d* of the ionomer layer can be estimated from the integration of the variable current during the CV experiment using Faraday’s law (assuming a single Faradaic reaction):

(3) $$d=\frac{\int_{0}^{t}i\left(t\right)dt}{F}\cdot \frac{M}{\rho \cdot A}$$

*F* is the Faraday constant (*F* = 96,485 C/mol), *M* is the molar mass of the PVBTMA repeat unit (211.6 g/mol), *ρ* is the PVBTMA density (1.3 g/cm^3^ [31]), and *A* is the Zn electrode area (1.7 cm^2^). From the experiment shown in Figure 1, the integrated charge is 2.8 C, and an ionomer film thickness *d* = 28 μm is obtained from Equation (3). The micrometric determination (Figure 1b) gives (30 ± 1) μm, which supports the integrated charge calculation and shows a high Coulombic efficiency of the electropolymerization process.

**Figure 3:**
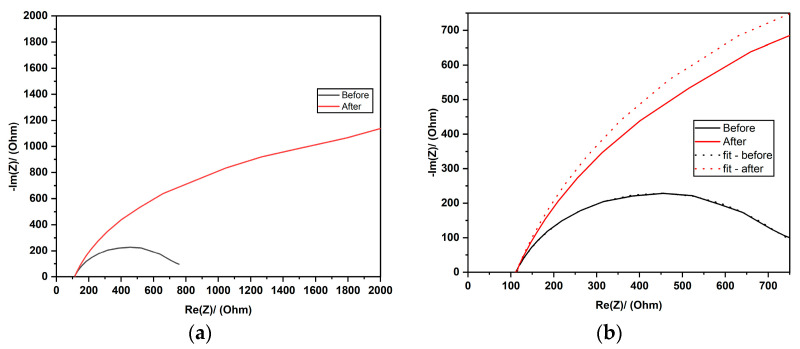
(**a**) Typical impedance spectra and fit before and after PVBTMA electropolymerization on Zn (precursor solution: 0.01 M); (**b**) zoomed-in view of the high-frequency region.

Typical impedance spectra before and after the electropolymerization of VBTMA are reported in Figure 3. The spectra are well-fitted by a Randles equivalent circuit of a resistance R1 in series with a parallel circuit of a resistance R2 and a constant-phase element Q2 (*CPE*). The impedance of a *CPE* can be written (*j* is the imaginary unit, and *ω* is the angular frequency):

(4) $$Z\left(CPE\right)=\frac{1}{Q}\cdot (j\omega {)}^{-n}$$

The modulus *Q* and the exponent *n* (0 < *n* < 1) are frequency independent; the case *n* = 1 describes an ideal capacitance while the case *n* = 0 corresponds to a pure resistance. The non-linear least-squares fit parameters are shown in Table 1.

Before the electrodeposition of PVBTMA on Zn, R1 corresponds to the resistance of the monomer solution in DMSO; the small increase after electropolymerization can be attributed to the PVBTMA coating. From the average resistance increase of 3 Ω and the coating thickness of 28 μm estimated from the cyclovoltammogram (Figure 1), the ionic conductivity of PVBTMA in a ClO_4_^−^ form (anion of the supporting electrolyte) can be calculated; the value (0.5 mS/cm) is consistent with data previously reported in the literature [30,31]. The resistance R2 increases strongly after electropolymerization. From the corresponding Q2 value, which has values in the order of interfacial capacitances, one can infer that R2 represents the charge transfer resistance; the strong increase is coherent with the protection of the Zn electrode against corrosion processes leading to Zn dissolution. The large increase in the total resistance R1+R2 is consistent with the continuous decrease in the cathodic current observed during the electropolymerization (Figure 1).

**Figure 4:**
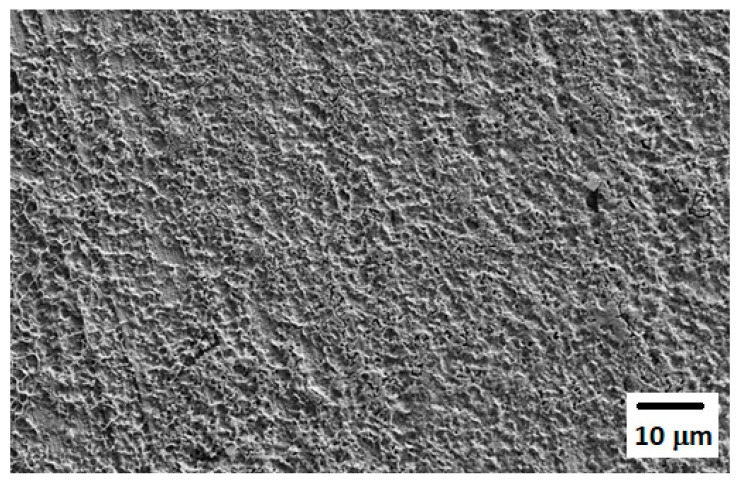
SEM micrograph of Zn disk with PVBTMA layer.

SEM micrographs of zinc pellets coated by electropolymerized PVBTMA show a rough surface (Figure 4).

**Figure 5:**
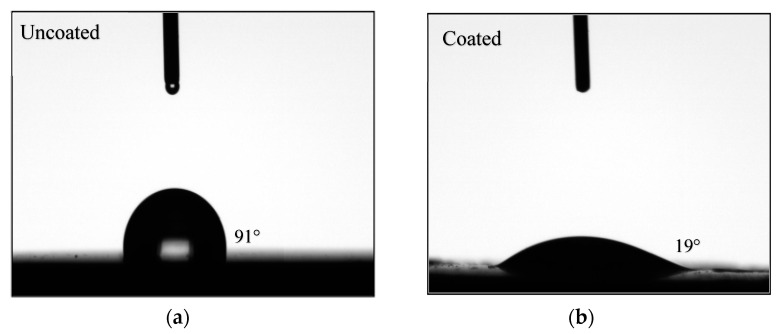
Contact angle measurements: (**a**) bare Zn disk; (**b**) Zn with PVBTMA coating.

The surface of the uncoated zinc disk shows a contact angle of 91° (Figure 5a). The much lower contact angle of 19° for the PVBTMA-coated sample (Figure 5b) is consistent with the high hydrophilicity of the ionomer due to the presence of hydrated nanometric channels [35]. It guarantees good wetting by the aqueous alkaline solution in the Zn–air battery.

### 3.2. ZAB Cycling Tests in Alkaline Solution

**Figure 6:**
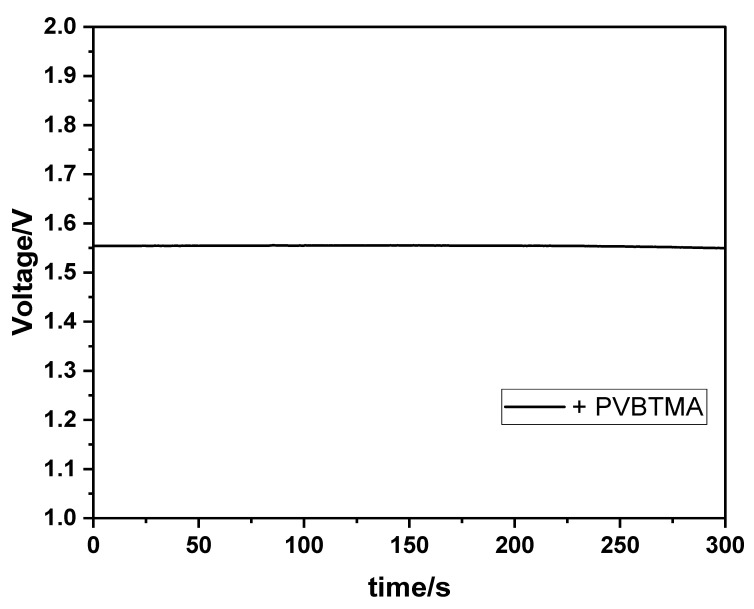
Open-circuit voltages of ZAB with PVBTMA-coated Zn.

The open-circuit voltage (OCV) of a ZAB with a PVBTMA-coated Zn anode is quite high (Figure 6). The OCV value is very similar with different PVBTMA thicknesses. The OCV is related to the electrocatalyst for the ORR because the ratio of four-electron and two-electron reduction can be modified. With the Pt counter electrode used in a liquid electrolyte, the four-electron mechanism is predominant, and the open-circuit voltage is near the theoretical value (≈1.6 V) obtained from Equations (1) and (3).

**Figure 7:**
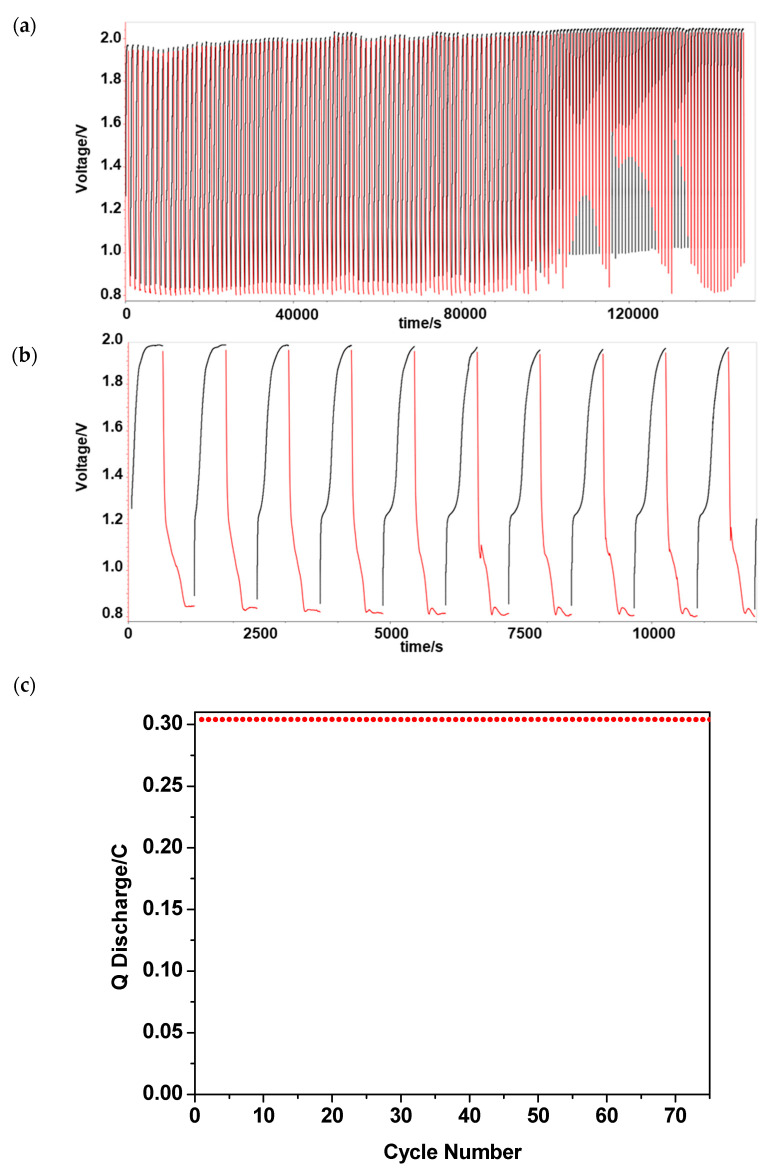
(**a**) Charge–discharge curves (150 cycles) of ZAB with PVBTMA-coated Zn (0.01 M precursor solution) in alkaline electrolyte with i = 0.5 mA/cm^2^, (**b**) zoomed-in view of the first 10 cycles and (**c**) discharge capacity vs. cycle number.

Figure 7a shows the charge–discharge curves of a ZAB with PVBTMA-coated Zn over 150 cycles with a current density of 0.5 mA/cm^2^. A zoomed-in view of the first 30 and 10 cycles is shown in Figure 7b,c, respectively. One observes very good cyclability over the first 95 cycles, and then a degradation of the ZAB is observed.

**Figure 8:**
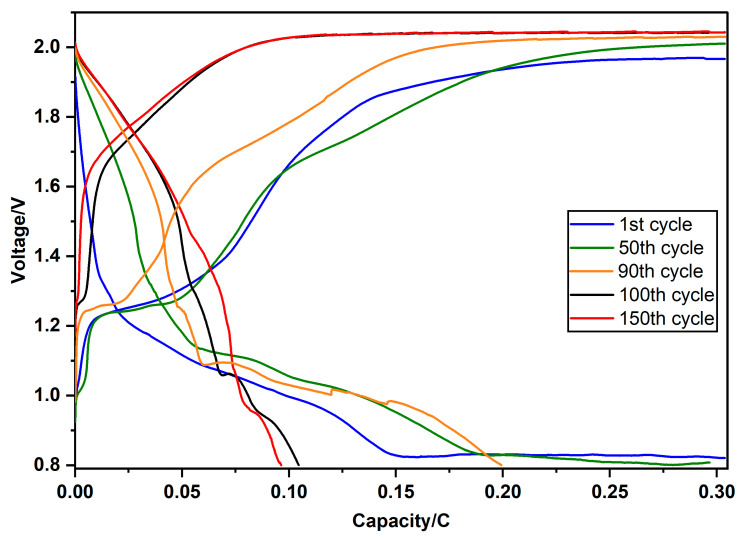
Galvanostatic charge–discharge profiles of ZAB in alkaline electrolyte (i = 0.5 mA/cm^2^) with PVBTMA-coated Zn anode as function of the cycle number.

Voltage–capacity curves for uncoated and PVBTMA-coated Zn anodes are reported in Figure 8. On charging, a plateau around 1.3 V can be seen, indicative of a biphasic equilibrium, probably involving Zn and ZnO, before further polarization until the final plateau around 1.95 V; the upper voltage limit of 2.1 V is not reached. The plateau around 1.3 V gets gradually shorter with cycle numbers until it disappears after 95 cycles concomitant with a clear degradation of the discharge capacity (Figure 8), probably related to a progressive blocking of Zn diffusion near the anode, as evidenced by impedance spectroscopy (see below). The discharge profiles exhibit sloped lines without a plateau; a pseudocapacitive response is observed slightly above 0.8 V. The discharge capacity decreases progressively with the cycle number. A clear profile change is observed above 90 cycles.

**Figure 9:**
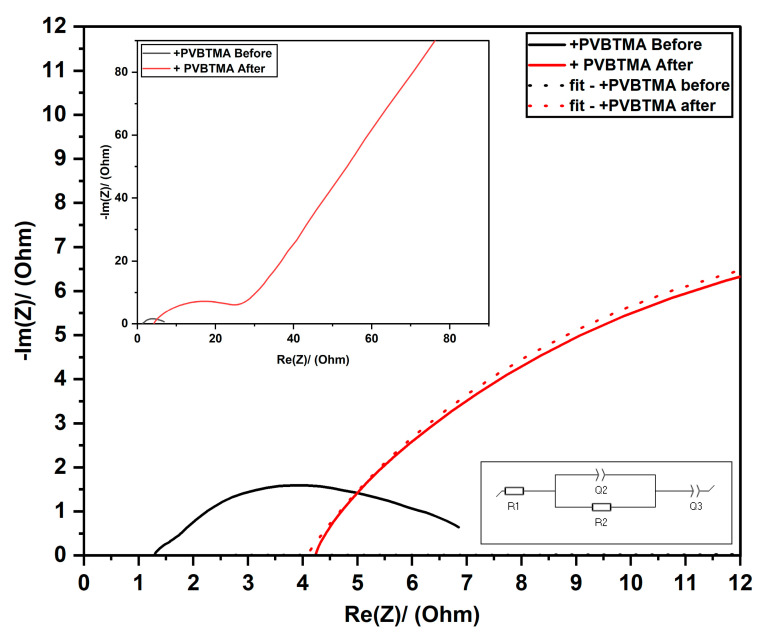
Impedance spectra before and after cycling of ZAB in alkaline solution using bare Zn and Zn with PVBTMA coating (insets: zoomed-in view (**top**), equivalent circuit (**bottom**)).

Typical impedance spectra before and after cycling are shown in Figure 9 for PVBTMA-coated Zn anodes. A second CPE Q3 must be added to describe the low-frequency behavior. The corresponding best-fit parameters are reported in Table 1. After cycling, the charge transfer resistance is further enhanced. Q3 evolves versus a capacitive behavior with a significant enhancement of the n3 value. Both results can be attributed to a progressive blocking of Zn diffusion near the anode, reducing the cyclability of the ZAB.

**Figure 10:**
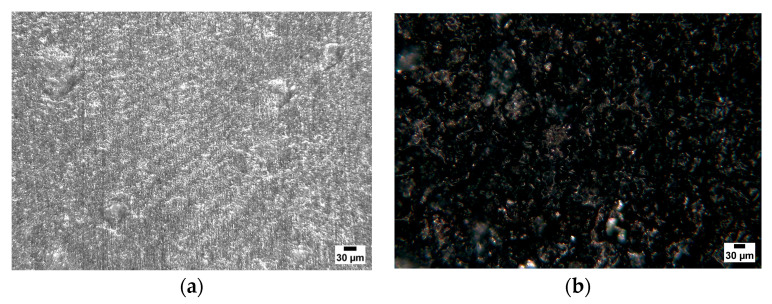
Optical microscope images of a Zn anode with PVBTMA coating: (**a**) before cycling; (**b**) after 150 cycles.

The most prominent observation from the optical imaging of PVBTMA-coated Zn anodes is the darkening of the surface of the zinc electrode after cycling (Figure 10). This occurrence can be ascribed to the zinc deposits on the surface.

Carbonate formation from the electrolyte can also be observed from white spots. It can be inferred from the PVBTMA-uncovered spots on the pellet circumference that the ionomer coating improved the homogeneity of the Zn deposition on charge. In a forthcoming article, the properties of symmetric Zn/Zn cells with bare zinc and PVBTMA-coated Zn will be analyzed to shed more light on the protection mechanism [36].

### 3.3. Miniaturized ZAB Tests

**Figure 11:**
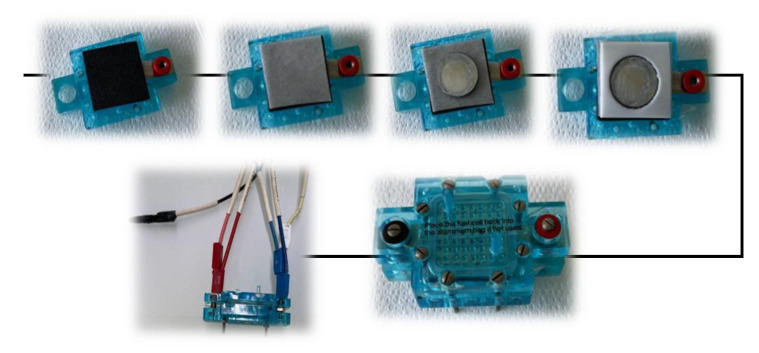
Miniaturized zinc–air battery components and assembly. The images show from the left the cathode part with carbon paper, the Whatman paper soaked with electrolyte, the PVBTMA-coated zinc anode and a Teflon piece to provide a robust assembly.

The “solid-state” configuration of ZABs is more practical for real-world use. Figure 11 shows the components and assembly process of the miniaturized zinc–air battery. For the assembly, we modified a teaching fuel cell (Fuel Cell Store) by removing the membrane electrode assembly, closing the H_2_ inlet and keeping the current collectors.

**Figure 12:**
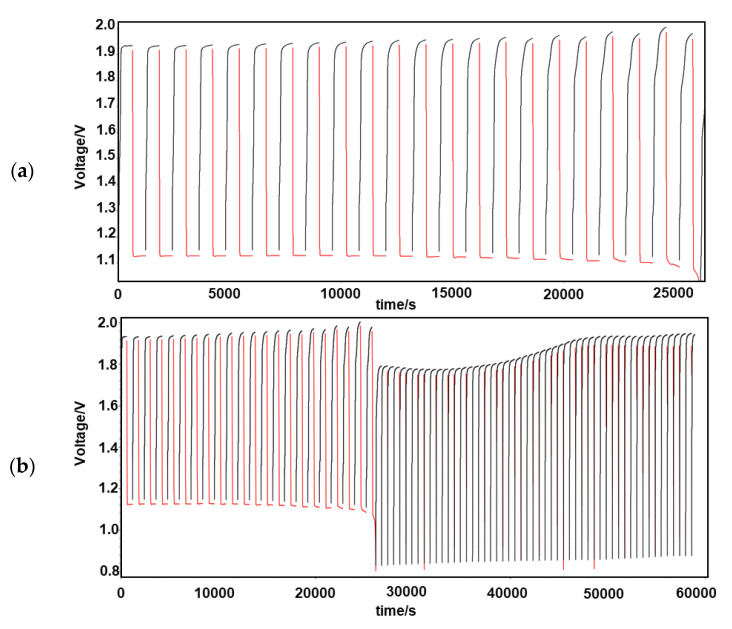
(**a**) First 22 charge–discharge cycles of miniaturized ZAB with PVBTMA-coated Zn; (**b**) ZAB charge–discharge cycling (75 cycles).

Figure 12 shows a cycling test of a miniaturized ZAB with PVBTMA-coated Zn via chronopotentiometry at 0.5 mA/cm^2^. One notices stable charge–discharge cycles within set voltage limits for 22 cycles followed by a sudden breakdown which might be related to various causes, including the clogging of the air electrode by precipitated carbonate or the drying out of the electrolyte sorbed on the Whatman paper.

**Figure 13:**
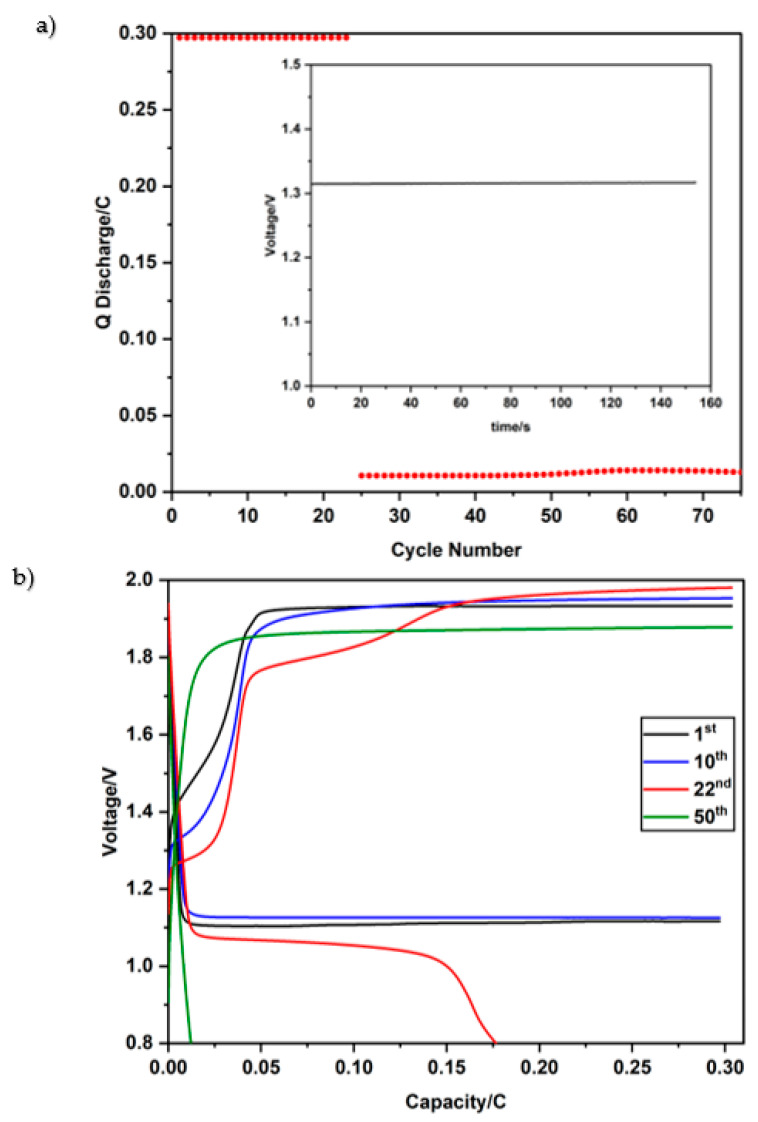
(**a**) Discharge capacity vs. cycle number (red dots) and open-circuit voltage (inset) and (**b**) voltage versus capacity curves at various cycles for a miniaturized ZAB with PVBTMA-coated Zn.

The measured open-circuit voltage of the ZAB was about 1.31 V (Figure 13a inset), a value somewhat lower than that in the liquid state. The open-circuit voltage is related to the used metal-free carbon paper electrode, at which the ORR proceeds predominantly via the two-electron mechanism [14] with the formation of hydroperoxide anions according to the following reaction:

(5) $$O_{2}+2\;e^{-}+H_{2}O\;⇆\;{{HO}_{2}}^{-}+{HO}^{-}\;\;\;\;E{}^{\circ}=-0.07\;V$$

The theoretical voltage for two-electron reduction, calculated from Equations (1) and (5), is of the order of 1.2 V; the slightly higher experimental value indicates that some four-electron reduction is also taking place. The other significant difference is the smaller potential difference of 0.85 V compared to 1.1 V in the liquid state, which can be also related to the different positive electrode material.

**Figure 14:**
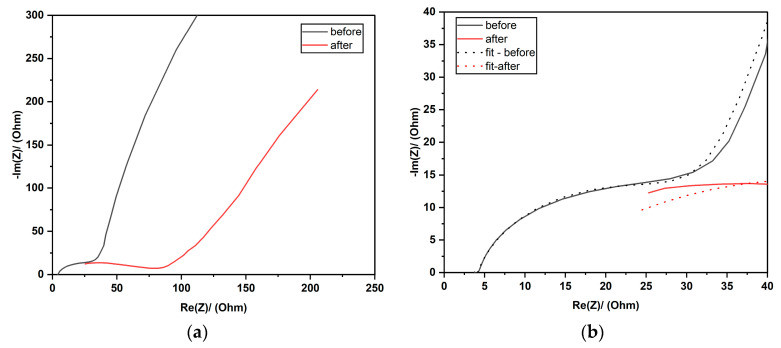
(**a**) Typical impedance spectra of miniaturized ZAB with PVBTMA-coated Zn and (**b**) zoomed-in view with Zplot fit.

The comparison with a ZAB in an alkaline solution shows a faster capacity decrease during cycling which can be attributed to the drying out of the electrolyte soaked in the Whatman paper of the miniaturized battery setup. The pores of the cathode can also be clogged by carbonate formation. From Figure 13b, the initial part around 1.3 V is followed by a slope before gradually ending with a longer plateau whose voltage slightly increases until the 22nd cycle up to 1.95 V. Altogether, the miniaturized ZAB results are promising [37]. Typical impedance spectra before and after cycling are presented in Figure 14. These spectra are consistent with the previous discussion of the alkaline electrolyte. The evolution of the exponent n2 toward the Warburg element value (0.5) indicates a growing diffusion limitation after cycling.

## 4. Conclusions

Zinc–air batteries (ZABs) are rechargeable batteries with a bright future, due to their good electrochemical performance, especially their high capacity, their safety, the absence of toxic products and their low cost. However, several important challenges must be overcome: the principal problems concerning the negative electrode are the corrosion problems of zinc in alkaline solutions with the formation of hydrogen gas and the growth of zinc dendrites during recharging. Both problems can be mitigated by an appropriate anode protection layer. In this work, the zinc protection layer was synthesized by the direct electrochemical deposition of the anion exchange ionomer poly(N-vinylbenzyl N,N,N-trimethylammonium) chloride (PVBTMA). An ionomer layer around 30 μm was obtained with a monomer concentration of 0.01 M. Major advantages of this ionomer are the excellent interface quality due to the in situ electrodeposition process, its anion selectivity and the high stiffness of its polystyrene-type backbone. The ZAB tests with a PVBTMA-coated Zn anode in an alkaline solution show very good cycling performance for nearly 100 cycles. The cycling tests of a miniaturized ZAB with PVBTMA-coated Zn show good performance for more than 20 cycles before a sudden breakdown, probably related to the clogging of the air electrode by carbonatation and the drying out of the electrolyte sorbed in the Whatman paper. The optimization of the ionomer protection layer appears to be an appealing strategy for future improved ZABs.

## Figures and Tables

**Table 1:** Non-linear least-squares fit parameters before and after PVBTMA coating deposition on Zn and before and after cycling in alkaline solution and in miniaturized batteries.

Parameters	Bare Zn Electrode (Before Electro-Deposition)	PVBTMA-Coated Zn Electrode (After Electro-Deposition)	Liquid Electrolyte ZAB		Miniaturized ZAB	
			PVBTMA-Coated Zn Anode before Cycling	PVBTMA-Coated Zn Anode after Cycling	PVBTMA-Coated Zn Anode before Cycling	PVBTMA-Coated Zn Anode after Cycling
R1/Ohm	108.0	111.0	1.3	4.1	4.1	12.8
R2/Ohm	636.0	2257.0	5.3	22.1	27.7	67.0
Q2/μF.s^(n−1)^	9	7	1667	249	233	22
n2	0.77	0.81	0.68	0.68	0.82	0.53
Q3/mF.s^(n−1)^	-	-	-	7.9	2.1	5.6
n3	-	-	-	0.68	0.85	0.60

## Data Availability

Data are contained within the article.
